# Review of new regulations for the conduct of clinical trials of investigational medicinal products

**DOI:** 10.1111/j.1471-0528.2007.01415.x

**Published:** 2007-08-01

**Authors:** SS Bollapragada, JD Norrie, JE Norman

**Affiliations:** a Division of Developmental Medicine, Maternal and Reproductive Medicine, University of Glasgow, Glasgow Royal Infirmary Glasgow, UK; b Centre for Healthcare Randomised Trials (CHaRT), Health Services Research Unit, Aberdeen University Aberdeen, UK

**Keywords:** Clinical trial regulations, EU directive, good clinical practice

## Abstract

The EU Clinical Trials Directive came into force on 1 May 2004 and has changed the face of Clinical Trials of investigational medicinal products in the UK. An enthusiastic registrar or consultant who comes up with an idea for a therapeutic intervention now needs to comply with a complex and demanding set of legal, ethical and regulatory requirements, contravention of which may lead to criminal proceedings. The aim of this review was to detail the relevant procedures and regulations and to provide a ‘user-friendly’ guide to obstetricians and gynaecologists wishing to conduct a clinical trial of an investigational medicinal product. Sources of further information are listed.

## Introduction

On 1 May 2004, new regulations came into force across Europe to better protect the rights, safety and wellbeing of patients participating in clinical trials of investigational medical products (IMPs). This EU Clinical Trial Directive 2001/20/EU^1^ relates to the implementation of Good Clinical Practice (GCP) in the conduct of clinical trials of IMPs. This has been incorporated into UK law by the Medicines for Human Use (Clinical Trials) Regulations 2004.^2^ The EU has recently (July 2006) issued guidance on GCP issues.^3^ Anyone designing or conducting a clinical trial of an IMP has to now comply with these regulations.

The Medicines and Healthcare products Regulatory Agency (MHRA) provides a guide to what is a medicinal product.^4^ An investigational medicinal product is a pharmaceutical form of an active substance or placebo being tested, or to be tested, as a reference in a clinical trial. For example, oxygen that is used for respiratory support is considered as a medicinal product by the MHRA. Presently, only trials involving IMPs fall under the scope of the directive and are the focus of this review. Trials not involving IMPs (e.g. trials of surgical interventions) are excluded from this review, although the expectation of the stakeholders (sponsors, ethics committees, research and development (R&D) offices, the medical research community and participants) is that they would be conducted to the same standard, since patient safety and scientific robustness is important in all trials.

‘GCP’ is a set of rules and regulations^5^ by which clinical trials must be conducted. GCP was developed by regulatory authorities from USA, Europe and Japan at the International Conference of Harmonisation in 1997, and the main principle is that ‘the rights, safety and wellbeing of the trial subjects are the most important considerations and should prevail over the interests of society’.

It is therefore imperative that anyone who is involved in a clinical trial is not only qualified for the purpose but also trained in GCP. The UK Clinical Research Network, launched in February 2005, has training as an important part of its remit (www.ukcrn.org.uk/index/training).^6^

## The definition of a clinical trial

A ‘clinical trial’ is specifically defined by the EU Directive as any investigation in human subjects intended:

1. To discover or verify the clinical, pharmacological or other pharmacodynamic effects of one or more medicinal products.
2. To identify any adverse reactions to one or more such products.
3. To study absorption, distribution, metabolism and excretion of one or more such products with the object of ascertaining the safety or efficacy of those products.

The MHRA (the ‘Competent Authority’ for the UK) have released an algorithm to establish whether a study qualifies as a clinical trial under the EU Directive.^7^ If it does, then a Sponsor must be identified and a EudraCT number must be obtained, followed by authorisation from MHRA and approval by a Research Ethics Committee (REC) and the NHS R&D department. These steps can be performed in parallel.

## Protocol, Sponsor, authorisations and registration

### The protocol

A protocol is a full description of the aims and methods of the clinical trial. Under the UK Regulations, the protocol must now include the information listed in the guideline for GCP.^5^ The regulations require that the clinical trial has to be conducted in accordance with the protocol. The Sponsor must notify any ‘substantial amendment’ to the protocol both to the MHRA and to the main REC for approval. A user-friendly web-based protocol development tool, focusing on pragmatic trials, has been written by the EU funded Pragmatic Randomized Controlled Trials in HealthCare (PRACTIHC) research group and is available free at www.practihc.org.

### Finding a Sponsor

Since 1 May 2004, it is illegal to start a clinical trial without a Sponsor, defined by the EU Directive as an individual, company, institution or an organisation which takes responsibility for the initiation, management and/or financing of a clinical trial. A very useful guide on Sponsor responsibilities is given on the Medical Research Council/ Department of Health (MRC/DOH) generated Clinical Trials Tool Kit (www.ct-toolkit.ac.uk, under Planning a New Trial > Identifying a Sponsor).^8^

For commercially funded trials, the Sponsor will invariably be the drug company. For publicly funded trials (of, for example, head to head comparisons of licensed drugs), the funder (often a government agency or a charity) usually look to the employer of the Chief Investigator—typically the NHS or a University, or both—to be the Sponsor.^9^ Although the Chief Investigator can act as Sponsor, their employer is much better placed to cover the risks in a clinical trial through their institutional indemnity and insurance policies, a key aspect to gaining financial approval for the study. It is important to secure sponsors early in the planning process and have written, legally binding contracts detailing the responsibilities that have been agreed between the partners. The R&D department of either the University or the NHS trust can give guidance on regulatory requirements and instigate a risk assessment of the study. The NHS R&D Forum^10^ (www.rdforum.nhs.uk) gives general information about the roles and functions of NHS R&D departments.

### Getting authorisations (MHRA, Ethics and R&D)

#### MHRA approval

The European Directive requires the Sponsor to obtain authorisation from the Licensing Authority of the Member State in which it is planned to conduct the trial (in the UK, the MHRA). This is facilitated at a ‘Sponsor interface’, located at eudract.emea.europa.eu, where a request for authorisation consists of the application form, supporting information (including protocol) and the applicable fee. The site also issues an obligatory EudraCT number uniquely identifying the trial. The MHRA has 30 days to object to the trial and give the grounds for nonacceptance (for full details, see http://www.mhra.gov.uk, under How we regulate > Medicines > Licensing of medicines > Clinical trials > How to submit a clinical trial application).

#### Ethics approval

All research in the NHS must be approved by a Department of Health recognised REC. Detailed information and pro formas are available on the Central Office for Research Ethics Committees (COREC) website (www.corec.org.uk). The ethics committee will balance risks to patients against expected benefit and will also review confidentiality and consent issues. Advice from an experienced investigator is invaluable in preparing an ethics application. Under the EU Directive and the Clinical Trials Regulations, a REC must give an opinion on a clinical trial within 60 days of receipt of a valid application and should reach one of four possible decisions: ‘Final opinion’—favourable or unfavourable; ‘Provisional opinion’ (with further information or revision required) or ‘No opinion’ (a referee needs to be consulted before an opinion can be given).

#### R&D approval

The relevant NHS R&D department must approve trial commencement at a local site, having assessed whether adequate provision has been made for the costs of NHS staff and consumables in the conduct of the study on NHS premises. Since January 2007, the R&D application form has been merged with part C of the REC form.

### Trial registration

Trials that begin enrolment of patients after 1 July 2005 must register in a recognised trials registry at or before the onset of enrolment to be considered for publication in most leading medical journals.^11^ Registration of trials is seen as an important safeguard against nonreporting of results.^12^ The two most commonly used are the US-based registry at www.clinicaltrials.gov and the European-based International Standard Randomised Controlled Trial Number registry at www.controlled-trials.com/isrctn.

## Conduct of a trial

### Study documentation

Every clinical trial is legally required to have a Trial Master File (TMF), containing all the essential documentation for all stages of the trial. It permits evaluation of the conduct of the trial and the quality of data produced.^13^ The TMF is maintained at the principal site (Chief Investigator’s office or Coordinating Centre) and should be accessible from all participating sites.

### Informed consent

The Chief Investigator has overall responsibility for the consent process. Consent must be informed and voluntary. The Chief Investigator must ensure that any member of staff who is involved in the process of obtaining informed consent is fully informed and trained to obtain it.

### Data management

Efficient data management is fundamental to any clinical trial. If data collected is incorrect or of poor quality, then the analysis and conclusions may be flawed. Trials need a robust system in place to ensure the accuracy and completeness of the data collected and its storage, increasingly provided by formal trials support units.

### Trial monitoring

The purpose of trial monitoring is to verify that the rights and wellbeing of human subjects are protected; the trial data are accurate, complete and verifiable from source documents; and that the conduct of the trial is in compliance with GCP. Site monitoring, central monitoring and scrutiny by a Data Monitoring Committee (DMC) can play a part in monitoring. Often a Sponsor will delegate responsibility for monitoring to the Chief Investigator.

#### Site monitoring

A person independent of the site, such as the trial manager, visits a trial site to check adherence to the protocol, to check informed consent documentation is complete and to verify events recorded on case report forms. In commercial trials, site monitoring tends to be very intensive and costly, as compared with publicly funded trials.^14^,^15^

#### Central monitoring

Central monitoring of data using statistical methods is increasingly being found useful in large multicentre trials for identification of unusual patterns of data. This allows scare monitoring resource to be targeted at sites in which problems may be emerging.

#### DMC monitoring

A DMC, independent of both Sponsor and investigators, is usually the only group that would expect to see analyses of the accumulating data by randomised group (i.e. with the ‘blind’ broken). Its main roles are to ensure that there are no safety issues why the trial should not continue or that there is such overwhelming evidence of benefit that to continue the trial would be unethical. The remit and operation of a DMC is thoroughly discussed by Grant *et al.*^16^

### Progress reports

The Chief Investigator must provide annual progress reports to R&D departments, the MHRA and the Ethics Committee, and possibly the funders. More frequent reports and meetings are considered good practice for the trial management group, comprising the investigators and study personnel.

### Pharmacovigilance

Clinical trials are generally designed to show efficacy, but they also document safety (such as potentially dangerous adverse effects of novel drugs). An ‘adverse event’ is any untoward experience affecting the health of a person recruited into the trial or any clinically relevant and potentially harmful laboratory result.

All trials require systems to report adverse events both rapidly (e.g. within a fortnight) and periodically (e.g. annually) in accordance with the legislations. Importantly, the investigator has to report any serious adverse event to the Sponsor, and the Sponsor has to report any suspected unexpected serious adverse reaction immediately to MHRA (in UK) and to the ethics committee. Detailed guidance is available on the collection, verification and presentation of adverse events.^17^

By collecting all safety data on trials in Member States (with a combined population of over 400 million) into a single European pharmacovigilance database, it is hoped that early identification of safety signals from clinical trials data will be possible.^18^

### Quality assurance and quality control systems

Statutory Inspections are systematic and independent examination of trial processes and documentation to determine whether the trial was properly conducted, analysed and accurately reported. In the UK, statutory inspections are the responsibility of the MHRA. In addition to statutory inspections, funders, sponsors and NHS R&D departments may want to conduct audits of trial sites.

## Publication issues

All trials should be written up and published regardless of whether the results are positive, negative or inconclusive.^19^ A report of a randomised controlled trial should convey to the reader, in a transparent manner, why the study was undertaken and how it was conducted and analysed. The CONSORT statement is an important research tool that takes an evidence-based approach to improve the quality of reports of randomised trials.^20^ In addition, there is now a move by the public funders of research to support an open access model for publishing. This has potentially significant consequences for the way in which research is published.^21^

## Study closedown and archiving

The protocol should clearly define the end of a trial, usually the completion of recruitment and the appropriate follow-up period. The competent authority (MHRA), ethics committee and R& D department must be notified within 90 days of a trial ending (15 days if it is terminated early, with the reasons for early termination).

Essential documents must be archived and be accessible for audit and inspection by regulatory authorities. The EU Directive requires retention for at least 5 years after trial end (for nonregulatory submission trials) and for a trial leading to a product licensing submission, at least 2 years after the last approval of a marketing application in the EU. In publicly funded trials, researchers are increasingly using reliable and affordable scanning technology to digitise their key documents, storing research documents indefinitely at little continuing cost. However, care needs to be taken to ensure that such digitised documents are kept in an established data archive so that they can be migrated, without loss of any data, from one system to the next as the information technology changes. In addition, there is an important trend to make available the raw study data, after an appropriate time, in publicly accessible data archives, thus allowing the scientific community opportunity for further analysis and research.^22^

## Conclusion

With the new regulations in place, while the trials may appear very complex to conduct, in a way they are ‘easier’ as the process is now well described with clear guidelines for every step. However, these processes are time consuming and have to be followed in logical order. In summary, the first step is to confirm that the trial falls within the scope of the UK Regulations. If it does, then the trial protocol should be written and funding sought with the regulations in mind. A Sponsor must be identified and a EudraCT number obtained, followed by authorisation from MHRA and approval by an Ethics Committee and the NHS R&D department. During the conduct of the trial, at least annual progress reports are required to the stakeholders (including regulators, ethics, sponsors and funders). Safety reporting (both rapid and periodic) is key. Continuing monitoring of the trial to fulfil these progress reporting is now obligatory. Keeping accurate trial documentation for all stages before, during and after the conduct of a trial is important, and one should be prepared for external inspection of trial data and processes. Clinical research in the UK is becoming increasingly network orientated, with the establishing of the UK Clinical Research Collaboration (www.ukcrc.org). Although currently there are few formally established clinical networks in obstetrics and gynaecology (with perhaps the exception of gynaecological oncology), these networks are beginning to emerge, allied with the Royal College. Investigators wishing to develop a trial of an investigational medicinal product should seek the support of such networks and in addition need to involve an established clinical trials unit to assist both in gaining funding and in complying with the existing relevant regulations.

## Web resources

www.corec.org.uk/ Central Office for Research Ethics Committees (COREC). All information available for obtaining ethics approval for NHS related work.

www.ct-toolkit.ac.uk/ Extremely useful for guidance on practical implantation of EU Directive. Jointly created by DOH/MRC.

www.emea.europa.eu/ The European Medicines Agency (EMEA) is a European Agency for evaluation of medicinal products. Provides access to EudraCT system to apply for EudraCT number.

www.mhra.gov.uk/ Official site of the Medicines and Healthcare products Regulatory Agency (MHRA).

www.ich.org/ Official site of the International Conference on Harmonisation (ICH)

www.amrc.org.uk/ AMRC (Association of Medical Research Charities) is a membership organisation of the leading UK charities that fund medical and health research. It was founded in 1972 and established as a charity in 1987.

www.tmn.ac.uk/ The UK Trial Manger’s Network (UKTMN) is a forum for those running publicly funded trials.

www.ukcrc.org/ UK Clinical Research Collaboration (UKCRC). The UKCRC brings together the key organisations that shape the clinical research environment in the UK. This includes the main funding bodies, academia, the NHS regulatory bodies, industry and patients

www.practihc.org/ PRACTIHC is an EU funded international collaboration to develop open access tools for the design and conduct of pragmatic healthcare intervention trials, particularly in developing countries. The related Clinical Trials Simulator (available free at http://www.randomization.org) allows investigators to explore the properties of various potential designs.
